# Multiparametric Flow Cytometry for MRD Monitoring in Hematologic Malignancies: Clinical Applications and New Challenges

**DOI:** 10.3390/cancers13184582

**Published:** 2021-09-12

**Authors:** Giovanni Riva, Vincenzo Nasillo, Anna Maria Ottomano, Giuliano Bergonzini, Ambra Paolini, Fabio Forghieri, Beatrice Lusenti, Patrizia Barozzi, Ivana Lagreca, Stefania Fiorcari, Silvia Martinelli, Rossana Maffei, Roberto Marasca, Leonardo Potenza, Patrizia Comoli, Rossella Manfredini, Enrico Tagliafico, Tommaso Trenti, Mario Luppi

**Affiliations:** 1 Department of Laboratory Medicine and Pathology, Diagnostic Hematology and Clinical Genomics, AUSL/AOU Modena, 41124 Modena, Italy; vincenzo.nasillo@unimore.it (V.N.); ottomano.annamaria@aou.mo.it (A.M.O.); bergonzini.giuliano@aou.mo.it (G.B.); beatrice.lusenti@unimore.it (B.L.); enrico.tagliafico@unimore.it (E.T.); t.trenti@ausl.mo.it (T.T.); 2 Section of Hematology, Department of Medical and Surgical Sciences, University of Modena and Reggio Emilia, AOU Modena, 41124 Modena, Italy; paolini.ambra@aou.mo.it (A.P.); fabio.forghieri@unimore.it (F.F.); patrizia.barozzi@unimore.it (P.B.); ivana.lagreca@unimore.it (I.L.); stefania.fiorcari@unimore.it (S.F.); silvia.martinelli@unimore.it (S.M.); rossana.maffei@unimore.it (R.M.); roberto.marasca@unimore.it (R.M.); leonardo.potenza@unimore.it (L.P.); mario.luppi@unimore.it (M.L.); 3 Pediatric Hematology/Oncology Unit and Cell Factory, Istituto di Ricovero e Cura a Carattere Scientifico (IRCCS) Policlinico San Matteo, 27100 Pavia, Italy; pcomoli@smatteo.pv.it; 4 Centre for Regenerative Medicine “S. Ferrari”, University of Modena and Reggio Emilia, 41125 Modena, Italy; rossella.manfredini@unimore.it

**Keywords:** MRD, flow cytometry, immunophenotype, leukemia, myeloma, lymphoma, immunotherapy

## Abstract

**Simple Summary:**

In hematologic cancers, Minimal Residual Disease (MRD) monitoring, using either molecular (PCR) or immunophenotypic (MFC) diagnostics, allows the identification of rare cancer cells, readily detectable either in the bone marrow or in the peripheral blood at very low levels, far below the limit of classic microscopy. In this paper, we outlined the state-of-the-art of MFC-based MRD detection in different hematologic settings, highlighting main recommendations and new challenges for using such method in patients with acute leukemias or chronic hematologic neoplasms. The combination of new molecular technologies with advanced flow cytometry is progressively allowing clinicians to design a personalized therapeutic path, proportionate to the biological aggressiveness of the disease, in particular by using novel immunotherapies, in view of a modern decision-making process, based on precision medicine.

**Abstract:**

Along with the evolution of immunophenotypic and molecular diagnostics, the assessment of Minimal Residual Disease (MRD) has progressively become a keystone in the clinical management of hematologic malignancies, enabling valuable post-therapy risk stratifications and guiding risk-adapted therapeutic approaches. However, specific prognostic values of MRD in different hematological settings, as well as its appropriate clinical uses (basically, when to measure it and how to deal with different MRD levels), still need further investigations, aiming to improve standardization and harmonization of MRD monitoring protocols and MRD-driven therapeutic strategies. Currently, MRD measurement in hematological neoplasms with bone marrow involvement is based on advanced highly sensitive methods, able to detect either specific genetic abnormalities (by PCR-based techniques and next-generation sequencing) or tumor-associated immunophenotypic profiles (by multiparametric flow cytometry, MFC). In this review, we focus on the growing clinical role for MFC-MRD diagnostics in hematological malignancies—from acute myeloid and lymphoblastic leukemias (AML, B-ALL and T-ALL) to chronic lymphocytic leukemia (CLL) and multiple myeloma (MM)—providing a comparative overview on technical aspects, clinical implications, advantages and pitfalls of MFC-MRD monitoring in different clinical settings.

## 1. Introduction

Since the last decades of the past century, the development of affordable and sensitive methods to identify rare leukemic cells, still detectable—at very low levels—either in the bone marrow (BM) or in the peripheral blood (PB) of hematologic patients on post-treatment follow-up, has provided a striking improvement of our ability to quantify the resistant/recurrent disease burden, far beyond morphologic remission (i.e., below the conventional microscopy threshold of 1 out of 100 cells). Such a valuable notion of “Minimal, or Measurable, Residual Disease” (MRD) was rapidly turned into a fundamental prognostic parameter, with an emerging pivotal role in therapy decision-making. To date, MRD monitoring is highly recommended in various settings of hematologic malignancies, not only as strong independent post-diagnosis prognostic factor, but also as the best tool to guide risk-based therapeutic strategies. This approach follows the idea to target residual neoplastic cells before overt clinical relapse, and, potentially, to cure patients by eradication of resistant cancer stem cells.

**Figure 1:**
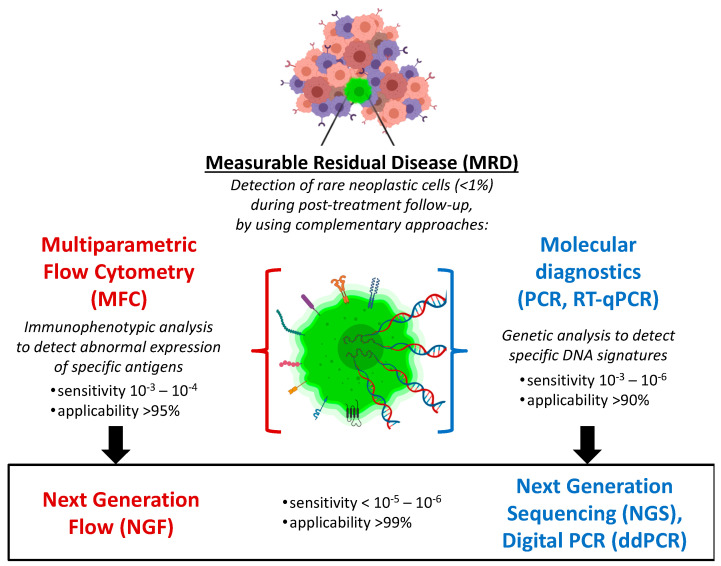
Complementary immunophenotypic and molecular approaches for hematologic MRD monitoring. MRD has widely emerged as the main post-treatment prognostic factor in different hematologic malignancies. Recent high-throughput evolutions of such advanced diagnostics are pushing forward the sensitivity, applicability and reproducibility of MRD detection, thus fostering new valuable integrations with clinical management and, in particular, with novel immunotherapeutic strategies.

Two complementary diagnostic approaches are currently used to perform, at diagnosis, an in-depth genomic and phenotypic characterization of tumor clones, and then, during clinical remission phase, to consistently provide accurate MRD quantifications, based on fine detection of tumor-specific abnormal features identified in each case. While molecular diagnostics pursue the characterization of *tumor genome* (aiming to disclose peculiar genomic lesions of the neoplastic clones), in parallel, flow cytometry allows the profiling of *tumor phenotype,* highlighting specific neoplasia-associated antigenic profiles (Figure 1). At present, molecular diagnostics is moving from qualitative PCR and real-time quantitative PCR (RQ-PCR) towards advanced PCR techniques (such as droplet digital PCR) and whole genome sequencing methods (the so-called “next generation sequencing”, NGS). Similarly, immunophenotypic analyses have also shown important progress, regarding both technical and interpretative aspects, evolving from basic (4-color) flow cytometry to multidimensional cell analyses (≥6–8 colors), generally known as “multiparametric flow cytometry” (MFC). Today, MFC-based MRD detection is based on the simultaneous recognition of several phenotypic markers (usually 6–8 antigens), as well as on the capacity to analyze big numbers of cells in few minutes, thus showing detection limits not far from those provided by the most sensitive molecular techniques. In perspective, a further step forward can be represented by “next generation flow cytometry” (NGF), which consists in a substantial improvement of high-throughput flow approach, allowing to rapidly acquire several millions of cells (>10^7^), and thus, reaching the sensitivity of molecular methods (10^−6^). MFC-MRD analysis can also take advantage of innovative software tools for automated gating (separation) of significant population (APS), as well as for assisted analysis of maturation pathways, in order to provide accurate and reproducible results, using up to 18 colors. Despite such technological and computational improvements, specific competences are still required to provide reliable and accurate MRD evaluation by MFC method, and such difficulty is still limiting the feasibility of MFC-MRD analysis outside few highly specialized laboratories.

In the last two decades, following the important technological progresses in cell acquisition and multiparametric analysis, big efforts have been done in order to overcome classic MFC limitations, in terms of reproducibility and comparability. Comprehensive recommendations on immunophenotypic analysis of hematolymphoid neoplasms were initially proposed by Clinical Cytometry Society in the 2006 Bethesda International Consensus Conference [1]. A few years later (in 2012), the EuroFlow consortium, aiming to improve standardization and guide the development of MFC-based diagnostics, provided novel consensus protocols, which redefined operative standards for MFC applications in the diagnostic process of hematologic diseases [2]. Based on EuroFlow guidelines, main international networks for hematologic neoplasms have recently elaborated specific procedural indications for sample collection, tube composition and multiparametric data analysis, as well as have suggested specific operational thresholds, useful to guide therapeutic interventions (Table 1).

In this overview, we summarize current recommendations for MFC-MRD monitoring in different hematologic malignancies, specifically discussing technical issues, therapeutic implications and new challenges emerging in each setting, also in comparison with molecular MRD diagnostics.

## 2. Acute Myeloid Leukemia (AML)

Acute myeloid leukemia (AML) is a heterogeneous neoplasia characterized by life-threatening outgrowth of BM myeloid progenitors, often requiring timely therapy intensification, based on the relapse risk of each patient after first-line induction treatment. The 2017 European LeukemiaNet (ELN) recommendations for AML included MRD assessment as a standard of care, MRD status now being required to define the deepness of treatment response (according to the new criterion “Complete Remission with/without MRD”) [3]. In 2018, the ELN MRD Working Party proposed five reasons to perform either immunophenotypic or molecular MRD diagnostics in AML: (i) to provide an objective methodology to establish a deeper remission status, (ii) to refine outcome prediction and inform post-remission treatment, (iii) to identify impending relapse and enable early intervention, (iv) to allow more robust posttransplant surveillance, and (v) to use as a surrogate end point to accelerate drug testing and approval [4].

According to ELN recommendations, MFC-MRD panels should include an essential set of surface markers, namely: early progenitor associated markers (CD34 and CD117), myeloid-lineage associated markers (CD11b, CD13, CD15 and CD33) as well as other differentiation markers (CD2, CD7, CD19, CD56 and HLA-DR,), with a backbone including CD34, CD117, CD13, CD15, CD33, CD7, always using CD45 gating and forward scatter/sideward scatter (FSc/SSc) plots. When required, a “monocytic tube” should be added, containing: CD4, CD11b, CD14, CD33, CD34, CD64, HLA-DR and CD45 [4]. Although formally not proven yet, last-generation instrumental setups using ≥8 colors (i.e., contextually investigating at least 8 immunophenotypic parameters on acquired cells) are expected to significantly improve specificity of MFC-MRD assays. During post-acquisition multiparametric analysis, the identification of the residual leukemic population can be optimized by taking advantage from two complementary approaches: (i) the former is based on the assessment of patient-specific “leukemia-associated immunophenotypes” (LAIPs) at the time of diagnosis, followed by a consistent tracking of such original LAIPs throughout post-therapy follow-up; (ii) the latter, named “different-from-normal (DfN) approach”, consists in a wider evaluation of abnormal differentiation/maturation patterns, possibly emerging during BM monitoring [4]. This latter type of advanced multiparametric analysis may usefully support classical LAIP-focused investigations, specifically helping to detect eventual modifications in the aberrant phenotype of leukemic cells, commonly referred as “immunophenotype shifts” (consisting in either gain or loss of antigens, due to clonal selection events, whose prognostic impact is still largely unknown). As a matter of fact, LAIPs themselves, which are characterized by either lineage infidelity or asynchronous expression of differentiation markers, can be seen as remarkable “DfN phenotypic signatures”, occasionally taking a specific diagnostic role. Hence, ELN MRD Working Party recommended to apply a combined “LAIP-based DfN approach”, where the specific LAIP tracking is well integrated into a broad immunophenotypic profiling of BM cells. Operatively, it is suggested to perform a full analysis of the above-mentioned markers, both at diagnosis and at follow-up, possibly adopting the same set of tubes and combinations of antibody/fluorochrome [4]. Of note, such a comprehensive MFC-MRD monitoring may allow to disclose some specific phenotype shifts that could offer the chance to exploit novel target treatments developed for other malignancies (as reported for the clinical case of a CD19-positive AML relapse, successfully treated with DLI infusions in combination with anti-CD19 blinatumomab [5], also in keeping with the use of such bispecific T-cell engager (BiTE) in patients with mixed phenotype acute leukemias [6]). 

Concerning the pre-analytical phase, adequate collection of BM samples remains a primary need to assure reliable quantification of MRD burden. Indeed, in order to accomplish this fundamental step, ELN guidelines specifically suggest using the first pull of BM aspirate and collect the lowest possible volume (ideally, 3–5 mL) in order to avoid the common pitfall represented by BM contamination with PB (since a dilution effect may negatively impact on measured percentages of leukemic cells). As a direct consequence of small BM volumes available for MFC-MRD analysis, a reasonable maximization of staining panels is advisable, aiming to use the lowest number of tubes, also considering that a high acquisition target (ideally, 1 million CD45+ leukocytes/tube) is required to fully exploit the performance of MFC-MRD diagnostics. Of note, at the beginning of post-acquisition analysis, it is encouraged to grossly estimate the level of BM sample contamination with PB (by evaluating the percentage of mature neutrophils found in each case). In keeping with this, the use of PB samples for MFC-MRD assessment is currently not recommended by ELN guidelines, in particular when low MRD levels are expected [4]. However, a recent work showed that PB MFC-MRD can be used as a biomarker for impending AML relapse [7], thus suggesting that some MFC-MRD applications could soon be validated also on PB samples, as already shown for molecular MRD monitoring.

With regard to clinical decision-making, ELN guidelines recommend to use a cut-off of 0.1% (10^−3^) for the operative definition of “positive” (≥0.1%) vs. “negative” (<0.1%) MFC-MRD values, as this threshold was found to be relevant in most MFC-MRD-guided clinical studies reported so far. However, although not formally yet validated, it should be taken into account whether, among MFC-MRD negative patients, MFC-MRD is undetectable (typically, <0.001%) or, rather, measurable (ranging 0.001–0.1%), by using current methods. Compared to molecular MRD [8], still little information is available on optimal timing for MFC-MRD assessment during AML follow-up: after most consolidated timepoints (i.e., post-induction and post-consolidation therapy), additional evaluations are commonly guided by the treatment protocol used. However, it could be advisable to perform MFC-MRD every 3 months, at least for the first 2 years of follow-up. Further studies are warranted (i) to investigate the prognostic values of MFC-MRD log10 modifications (as done for molecular MRD), as well as (ii) to shed light on the clinical impact of undetectable MFC-MRD, possibly identifying patients with very good prognosis. To date, while awaiting for clinical validations of promising NGS applications, molecular MRD monitoring is feasible only when distinct translocations or mutations are present (namely, PML-RARα, RUNX1-RUNX1T1, CBFβ-MYH11 and mutated NPM1), leaving MFC-MRD diagnostics to remain the only available method for monitoring the majority of AML patients, including the intermediate-risk group, typically characterized by the absence of genetic/molecular lesions [9].

Interestingly, in addition to the basic set of phenotypic markers, MFC-MRD monitoring can be extended to the investigation of other relevant antigens, which have shown some promising uses for diagnostic definition and prognostic assessment, as well as for therapeutic applications. Of note, a comprehensive MFC-MRD study reported a list of 22 markers aberrantly expressed in AML patients (namely, CD9, CD18, CD25, CD32, CD44, CD47, CD52, CD54, CD59, CD64, CD68, CD86, CD93, CD96, CD97, CD99, CD123, CD200, CD300a/c, CD366, CD371 and CX3CR1), which allowed to finely dissect normal vs. leukemic stem cell (LSC) pools among hematopoietic progenitors, thus providing a highly sensitive and specific tool for MRD monitoring (putatively, able to detect 1 leukemic cell out of >10^5^ normal BM cells) [10]. To date, CD123 and CD133 represent the most promising markers for LSC tracking (with a backbone including CD34, CD38, CD117, CD33, CD90, and CD45), and are also emerging as attractive targets for novel immunotherapies [11]. In particular, CD123 antigen (i.e., IL-3 receptor alpha chain) has attracted considerable attention: recently validated as main diagnostic marker for blastic plasmacytoid dendritic cell neoplasm (BPDCN), its expression has been reported in several cancers, including AML, myelodysplastic syndromes (MDS), myeloproliferative neoplasms, systemic mastocytosis, acute lymphoblastic leukemia (ALL), Hodgkin lymphoma, and hairy cell leukemia (HCL) [12,13]. In AML, CD123 was found to be significantly expressed by LSCs as well as by more differentiated leukemic blasts (but absent in the normal counterpart), and to correlate with the presence of FLT3/ITD-positive clones, thus representing a negative prognostic factor at diagnosis [14,15,16]. Different target treatments directed toward CD123-positive cells are under clinical investigations. Talacotuzumab (JNJ-56022473), a humanized anti-CD123 monoclonal antibody, showed ADCC-mediated cytotoxicity and direct inhibitory effects on IL-3-dependent tumor growth in AML xenograft murine models, but, so far, has failed to show a significant therapeutic advantage, either as a single agent or in combination with decitabine (possibly due to a lack of cell-mediated cytotoxicity in patients with advanced disease) [17]. It is conceivable that the therapeutic effect could be improved by the development of anti-CD123 antibody-drug conjugate (ADC) approaches (similar to the FDA-approved anti-CD33 gemtuzumab). In line with this idea, SL401 (tagraxofusp), a recombinant protein made of IL-3 fused to diphtheria toxin (already approved in 2018 for the use in BPDCN) [18], showed some promising results in a series of CD123+ AML/MDS patients [19,20]. Similarly, flotetuzumab (MGD006), a humanized bispecific dual-affinity re-targeting (DART) antibody, recognizing both CD123 on leukemic cells and CD3ε on T cells, displayed encouraging activity in adult patients with relapsed/refractory AML [21]. Moreover, CD133 antigen is well expressed by early hematopoietic progenitors and represents a common marker for LSCs, possibly implying an adverse prognostic significance [11]. Interestingly, CD133 is also a well-known primary marker of cancer stem cells in many tumor types, and has already been described as valuable target for novel chimeric antigen receptor (CAR) T cell therapies in solid cancer settings (such as hepatocellular, pancreatic and gastrointestinal carcinomas, as well as glioblastomas) [22], thus suggesting the opportunity to test this approach also against leukemic diseases [23]. In addition, C-type lectin-like molecule 1 (CLL-1), recently reported to be highly expressed in AML blasts, but typically absent in hematopoietic progenitors, has successfully been targeted by either BiTEs or CAR-T cells, in preclinical models of AML [24,25].

## 3. Chronic Myeloid Leukemia (CML)

In the setting of chronic myeloid leukemia (CML), which is invariably associated with BCR-ABL1 translocations (the so-called Philadelphia chromosome), MRD is classically monitored by RQ-PCR, which has been highly standardized across worldwide laboratories, and its prognostic role has fully been integrated with tyrosine kinase inhibitors (TKIs)-based therapeutic management. Notwithstanding, MFC-MRD diagnostics may be useful to identify and track the CML-LSC population (immunophenotypically identified by the co-expression of CD34, CD26, CD56, CD93, CD123, and lack of CD38), whose eradication represents the ultimate challenge in CML treatment [26,27,28,29]. Interestingly, a flow cytometry-based (but not immunophenotypic) immunobead assay for the detection of BCR-ABL1 fusion proteins in cell lysates was described. This protein-based approach, named “cytometric bead array (CBA)”, albeit rapid and easy, yields a low sensitivity (10^−2^), which limits its use in the clinical practice [30,31,32]. Moreover, a new experimental method, combing a molecular technique (namely, “in situ proximity ligation assay”) with flow cytometry as readout (PLA-flow), showed good sensitivity (10^−4^) and high correlation with BCR-ABL1 RQ-PCR, providing the opportunity to finely identify leukemic cells harboring BCR-ABL1 fusion proteins and, simultaneously, assess the expression of significative surface markers [33].

## 4. Acute Lymphoblastic Leukemia (ALL)

In ALL, which is characterized by aggressive outgrowth of BM lymphoblasts of either B-cell (B-ALL) or T-cell (T-ALL) origin, MRD is a fundamental prognostic factor, broadly used to define different risks of relapse and to guide clinical decisions, especially for treatment intensification by hematopoietic stem cell transplant (HSCT), both in adult and children setting [34,35,36,37,38]. Originally, seminal studies in pediatric ALL have represented a leading clinical experience of MFC-MRD use in hematologic patients [39,40,41,42,43]. Today, although RQ-PCR of Ig/TCR gene rearrangements and other specific gene fusions constitutes the most validated method for MRD monitoring in ALL, LAIP detection by MFC is commonly suggested to complement the molecular approach, as MFC can provide faster accurate results and deeper biological information [36,37,44,45,46,47].

In B-ALL, the specific challenge for MFC-MRD measurement is to phenotypically distinguish normal, regenerative B-cell precursors (BCPs, also known as hematogones, especially abundant in the BM during post-treatment follow-up), from leukemic populations of B lymphoblasts (i.e., neoplastic BCPs) [48]. Such pathologic clones can be commonly identified by abnormal expression patterns of classical BCP markers (namely, CD34, CD19, CD10 and CD20), and, sometimes, also by the acquisition of distinctive aberrant antigens (e.g., CD7, CD33 and CD58) [49]. In fact, a robust set of main BCP markers (CD34, CD19, CD10, CD20, plus CD38 and CD45) constitutes the backbone of the 8-color B-ALL MRD tubes proposed by main clinical studies, showing significant concordance between MFC and RQ-PCR approaches for B-ALL MRD monitoring [50]. Along with these six shared antigens, additional valuable markers (e.g., CD22 and CD81) were frequently included in the optimized tubes, while other B-ALL diagnostic markers, such as CD79a, TdT and CD24, were more often found to be poorly informative for MRD assessment. Mature B-cell markers, in particular surface immunoglobulins, are usually not recommended for MRD monitoring, as they do not help to differentiate normal versus leukemic BCPs. In particular, a recent multicentric EuroFlow study [36] has demonstrated that a fully standardized bulk lysis protocol with two stepwise designed 8-color tubes (including the BCP backbone panel plus CD81, and either CD66c/CD123 or CD73/CD304, combined in the PE fluorescence channel of each tube) allows highly sensitive MRD measurements (up to 10^−5^, comparable to PCR method, with >90% concordance) in virtually all B-ALL patients (>98%), as long as large numbers of events are acquired (>4 million BM cells). Of note, in the last years, the growing clinical use of innovative immunotherapies targeting CD19 (i.e., blinatumomab and CAR-T cells) and CD22 (inotuzumab ozogamicin) have suggested to extend MFC-MRD analysis to CD22 and CD24, which are expressed in early BCPs (even before CD19), and can be essential for tracking B-ALL clones with persistent downregulation of CD19 (frequently occurring after targeted treatments), as well as for the identification of CD22+ cases eligible for inotuzumab ozogamicin [51]. Thus, it is recommended that the MFC-MRD laboratory could always be informed on immunotherapies used in each case, in order to optimally set up patient-tailored MRD panels [36,51].

Clinical investigations on B-ALL prognostic factors have suggested that MFC-MRD can be defined as “negative” (complete MRD response) when <0.01%, while values ranging 0.01–1% are considered “positive” (MRD persistence/recurrence). However, by applying the NGF approach, it has been demonstrated that very low MRD levels (10^−5^ or less) can become detectable, i.e., reaching a sensitivity limit of molecular methods. In this view, the definition “undetectable MRD” should be specifically used to indicate the complete absence of neoplastic cells, at best of method sensitivity, while detectable, but not quantifiable (<0.01%), MRD levels (technically, “negative”) may actually represent an important “grey area” of warning. Most clinically relevant MFC-MRD timepoints remain: (i) early during induction treatment (typically, at day +15) and (ii) post-induction/consolidation therapy, both consistently allowing to identify either very good outcome patients (showing rapid tumor clearance), or poor outcome patients with persistent MRD, respectively [41,52,53]. In particular, such early prognostic stratifications are pivotal for the therapeutic choice about allogeneic HSCT transplant. Opposingly, long-term monitoring still lacks adequate standardization in B-ALL: timing and clinical uses of MFC-MRD follow-up are still unclear and commonly guided by the therapeutic protocol used. Here, again, MRD reappearance is typically diagnosed when ≥0.01% values are detected (and confirmed in a subsequent sample), but, in perspective, the evidence of low-level MFC-MRD recurrence (<0.01%) could be taken into account [54].

Compared to AML, also in B-ALL the requirement for BM representative samples is similar (or even more fundamental), while the dual analytic approach (LAIP- and DfN-based) is not strictly needed, since B-ALL is basically a less heterogeneous disease. However, also in this setting, a wider DfN-based analysis, including myeloid and T-cell antigens, may improve the identification of aberrant clones undergoing immunophenotypic shifts (with potential biological and therapeutic relevance), becoming particularly useful in patients diagnosed with mixed phenotype acute leukemia (MPAL) [55].

Contextually to MRD monitoring, MFC-based analyses may also promote the prognostic quantification of protective tumor-specific T-cell responses, naturally occurring in the BM or emerging after therapeutic T-cell infusions, as conceptually demonstrated in Philadelphia chromosome-positive (Ph+) B-ALL patients, showing inverse correlations between cytotoxic Bcr-Abl-specific/WT-1-specific T lymphocytes and MRD trends [56,57,58].

By considering T-ALL, analytic features and clinical applications of MFC-MRD have early shown many similarities with B-ALL setting [59]. In T-ALL too, a specific LAIP analysis is aimed to detect malignant lymphoblasts with abnormal expression of T-cell progenitor markers. However, the detection of immature T-cell precursors in either BM or PB is considered, per se, an abnormal finding [60], with PB MRD possibly conferring a higher risk of extramedullary relapse [61]. In detail, T-ALL backbone panels are set up to evaluate both the asynchronous expression of classic T-cell maturation markers (namely, membrane/cytoplasmic CD3, CD5, CD7, CD2, CD4, CD8, CD34, CD45) and the ectopic expression of thymic antigens (such as CD99 and CD1a), as well as TdT, CD10, CD38 and CD56 [50,62,63]. Recently, several works have consistently confirmed that MFC-MRD assay (performed early after induction therapy, by using 6–8 color panels with a threshold of 0.01%) well represents a consistent prognostic factor, both in adults and in children with T-ALL [64,65,66]. Further studies are needed to elucidate whether patients with early undetectable MFC-MRD can be eligible for reduced intensity therapy [67], and whether novel NGF approaches can actually improve MRD detection and clinical management in T-ALL patients [68].

## 5. Chronic Lymphocytic Leukemia (CLL)

Chronic lymphocytic leukemia (CLL), the most common leukemia in the Western world, is characterized by the clonal expansion of mature B cells in the BM, PB, spleen and lymph nodes. The diagnosis of CLL requires the presence of ≥5000/μL circulating clonal B lymphocytes, with a peculiar morphology and distinctive immunophenotype [69]. While for other hematological neoplasms (e.g., CML, ALL and AML) the use of MRD in daily clinical practice has been consolidated for several years, the history of MRD in CLL is rather young. Since the first publications proposing an assessment of residual disease (not so minimal at that time) with 2–3 color MFC panels [70,71], the concept of MRD, its applicability, and the detection assays have undergone profound changes. A historical breakthrough was made in July 2016, when the European Medicines Agency (EMA), after a public consultation with experts in the field, decided to include the assessment of MRD as an intermediate endpoint of phase III clinical trials for the approval of new drugs in CLL [72]. In CLL, indeed, the level of MRD after therapy (regardless of type of therapy) is an independent predictor of both progression-free survival (PFS) and overall survival (OS) [73,74]. Consistent with this, the use of MRD as a surrogate endpoint is expected to reduce the time necessary to assess the efficacy of the increasing number of drugs now available for CLL therapy. International workshop on CLL (iwCLL) [75], EMA [72] and Food and Drug Administration (FDA) [76] guidelines define MRD on the basis of the number of residual leukemic cells detectable in a sample of PB and/or BM compared to the total number of leukocytes. MRD is referred to as undetectable (U-MRD) if the residual proportion is <1 cell with CLL features among 10,000 leukocytes (10^−4^ or 0.01%). Therefore, MRD should be assessed by a standardized technique with a threshold lower than 10^−4^ [72,75,76]. A recent up-to-date expert review and consensus recommendations have innovatively proposed the introduction of “CML-inspired” categorical measures indicating the upper limit of MRD, i.e., MRD4 to identify cases with <10^−4^ MRD, MRD5 to identify cases with <10^−5^ MRD, etc., in order to provide additional information on assay sensitivity and whether disease is detectable below the reporting threshold [77]. Thus, the category MRD4 would indicate MRD <0.01%, but does not specify whether disease is detectable or not below this level [77]. However, while up to the 10^−4^ threshold MRD has an impact on survival with significant improvements in both PFS and OS per logarithm of reduction in tumor burden, no prospective data are currently available to support the idea that further reduction in MRD level (below 10^−4^) will provide additional clinical benefit. Moreover, according to guidelines: (i) MRD testing is not indicated in routine clinical practice, (ii) in clinical trials MRD should be evaluated in all responding patients, either in partial response (PR) or complete response (CR), at least in PB, (iii) U-MRD status documented on PB should be confirmed on BM, (iv) there is no specific recommendation to support the choice of a validated assay over another, since both MFC and molecular techniques (RQ-PCR, droplet digital PCR and high throughput sequencing) are considered adequate to assess MRD at the required threshold; hence, the assay choice depends on the rationale for MRD determination and local availability [75,77].

MFC assessment of residual CLL cells chiefly relies on the differential antigen expression between normal B cells and CLL cells. The presence of CD5 and CD23, along with a weaker expression of CD20, CD79b and CD81 compared to normal mature B cells, are the main distinguishing features of CLL cells. The first and simplest MFC panel for MRD evaluation in CLL, virtually applicable to all cases, albeit with a low sensitivity, was based on CD19/CD5 co-expression with demonstration of surface Ig light chain (κ or λ) restriction (as a surrogate marker of clonality). Over the years, the introduction of a growing number of antibodies in the MFC panel has allowed a more accurate detection of residual CLL cells. However, CD19/CD5/κ/λ combination can be used to screen samples with high burden of residual disease, in which a more extensive MFC panel is redundant [78]. Moreover, as CD20 shows a low intensity of expression in CLL, testing this antigen is helpful to discriminate between normal and CLL B cells. Nonetheless, rituximab-containing regimens can significantly downregulate the CD20 antigen, making it difficult to distinguish CLL cells from normal B cells. The introduction of CD79b has resulted in a 2-log increase of sensitivity compared to conventional 4-color analysis including CD19, CD5, CD20 and Ig light chain [79]. Furthermore, the addition of CD43 (homogeneously expressed on CLL cells) [80] to CD19/CD5/CD20 has allowed to reach a 100% specificity in detecting residual CLL cells, when compared to molecular assays, with a maximum sensitivity of 2.2 × 10^−4^ [81]. Similarly, the use of CD81, in combination with CD19/CD22/CD5, has warranted an accurate evaluation of MRD, also after anti-CD20 therapies, with a good specificity and sensitivity [82]. Since 2007, ERIC started to harmonize flow cytometry methods to assess MRD, in order to reduce the inter-laboratory variability, improve sensitivity and specificity, and allow comparison of results both among different clinical trials and different types of treatment [83]. The first standardized assays were based on 4-color (4 tubes) [83] and 6-color (2 tubes) [78] panels, both sensitive enough to define the absence of detectable MRD at the threshold recommended by iwCLL (i.e., 0.01%). Nevertheless, both testing strategies imply the need of dispensing the blood sample across multiple tubes, resulting in a prolonged acquisition time and impaired sensitivity in cases with poor cellularity. Since most new (8- or 10-color) flow cytometry instruments allow to combine the required antibody into a single tube, ERIC group has developed a core panel of six monoclonal antibodies (CD19, CD20, CD5, CD43, CD79b, CD81), which is applicable to >95% of typical CLL cases and is feasible in most laboratories [84]. The inclusion of both CD20 and CD22 is unnecessary in cases with typical expression of at least 2 markers among CD5, CD43, CD79b and CD81, while the addition of CD3 may be useful only if a very high accuracy in the 0.001–0.01% range is required [84]. Therefore, it can be concluded that the exclusion of CD3 and/or CD22 does not affect results below the limit of detection or above the 0.01% threshold. Further markers, such as CD160, CD200, CD23, and ROR1, likewise provide added value only in atypical cases or if an improved sensitivity (10^−4^–10^−5^) is needed [85,86,87]. According to this, the ERIC 6-marker core panel can be considered the most reliable and convenient assay to assess MRD in typical CLL cases, also being recognized as a standard test by regulatory agencies [84].

The progressive improvements in the quality of responses following the adoption of new therapeutic approaches have pointed out the need to measure increasingly lower MRD levels [88]. Nonetheless, the clinical relevance of U-MRD has been challenged in recent years with the advent of Bruton tyrosin kinase (BTK) inhibitors, particularly ibrutinib (the first-in-class BTK inhibitor) [89]. Despite their unprecedented efficacy allowing long-term control of the disease, BTK inhibitors rarely induce U-MRD as single agents, and a long-lasting lymphocytosis, not associated with an inferior outcome, is frequently observed in patients treated with ibrutinib alone, due to the redistribution of CLL cells into the bloodstream [90,91]. In contrast, the introduction of the first selective BCL-2 inhibitor (venetoclax) in the therapeutic armamentarium of CLL has clearly demonstrated that U-MRD can be achieved also by employing new targeted therapies within chemo-free regimens [92]. In particular, the efficacy of a fixed duration (2 years) treatment based on the combination of venetoclax and rituximab was investigated in a randomized phase III trial (MURANO) conducted on relapsed/refractory CLL patients. The updated results of the study not only confirmed a survival advantage in patients treated with venetoclax-rituximab compared to standard therapy (bendamustine-rituximab), but documented also very high rates of U-MRD on PB in the experimental arm (62%, compared to 13% in the standard treatment group) [93].

In conclusion, although current guidelines do not recommend routine MRD testing in clinical practice, it is conceivable that MRD status will be the key variable guiding the decision to halt or continue therapies with novel inhibitors.

## 6. Non-Hodgkin Lymphomas (NHLs)

The use of flow cytometry for MRD detection in non-Hodgkin lymphomas (NHLs) is not well established and is still a matter of investigations [94,95]. Molecular methods remain the mainstay for MRD monitoring in NHLs, especially in mantle cell lymphoma (MCL) and follicular lymphoma (FL) [94]. Moreover, for NHLs with predominantly nodal involvement, the circulating burden is typically too low to be detected by MFC, even at diagnosis. In addition, lymphomatous cells may lack unique immunophenotypic profiles, further limiting the applicability of MFC in this setting [96].

In MCL, although flow cytometry is considered a suitable diagnostic assay, no MFC-MRD panels have formally been validated for clinical purposes. A conventional 4-color panel, using surface Ig light chain restriction in the CD19+ CD5+ subpopulation, has been employed for MRD assessment in MCL, showing a significantly lower sensitivity (8 × 10^−4^) compared to RQ-PCR (10^−4^–10^−5^) [97]. However, even 8-color approaches, endowed with a better sensitivity (10^−4^), invariably remain inferior to molecular techniques, failing to detect about 20% of MRD-positive cases, as defined by using RQ-PCR [98,99]. In perspective, optimized MFC strategies as well as validation in the context of clinical trials are needed to fully implement MFC-MRD in MCL.

Hairy cell leukemia (HCL) is a chronic neoplasm of mature B cells, expressing some distinctive surface markers (i.e., CD103, CD25 and CD11c), which can be exploited for MRD evaluation, by using both immunohistochemistry (IHC) [100] and MFC [101]. In a monocenter experience, a single-tube 8-color assay was tested for MRD monitoring in 15 HCL cases, yielding a good sensitivity (10^−4^), comparable to RQ-PCR [101]. However, MFC-MRD applicability in HCL is typically limited by a lack of validation on large cohorts and by the technical difficulty of obtaining adequate amounts of leukemic cells from a frequently fibrotic BM. Since the BRAF V600E mutation is virtually present in all patients at diagnosis, mutation-specific droplet digital PCR has also been proposed as an innovative highly sensitive tool for MRD monitoring in HCL [102]. Of note, according to current HCL guidelines, MRD is still defined on the basis of leukemic infiltrate recognizable by IHC stains [103].

Compared to other NHLs, the role of MRD in the context of marginal zone lymphoma (MZL) is even less explored. Interestingly, the prognostic significance of MFC-MRD has recently been evaluated in 71 patients with splenic MZL treated with rituximab-based regimens. In this prospectively designed study, both 5-year PFS and 5-year OS were significantly higher in patients achieving U-MRD (<10^−4^ residual lymphomatous cells in the BM) than in MRD-positive patients, providing evidence for an independent prognostic role of MFC-MRD also in the setting of MZL [104].

## 7. Multiple Myeloma (MM)

Multiple myeloma (MM) is a malignant neoplasm of clonal plasma cells (PCs) growing in BM and usually producing a monoclonal protein, which can be represented by an intact immunoglobulin or a free light chain (FLC) [105]. These highly specific tumor markers are not only useful tools for clinical-laboratory monitoring, but also constitute the basis for classical response criteria [106]. In the last 10 years, the introduction of new agents, such as proteasome inhibitors, immunomodulatory drugs, and monoclonal antibodies, alongside autologous HSCT, have expanded the proportion of patients achieving high-quality responses (i.e., CR or stringent CR, sCR), which have been translated into prolonged PFS and OS [107]. Nevertheless, even patients in CR/sCR will ultimately experience the disease relapse, due to the persistence of residual clonal PCs in BM, which cannot be detected by conventional serological nor morphological methods, highlighting the need for more sensitive approaches [108]. The availability of highly sensitive techniques led to significant progress in detecting MRD both in BM, by using either MFC or molecular-based assays, and outside the BM, through metabolic or functional imaging techniques. Consequently, the International Myeloma Working Group (IMWG) updated the response criteria by incorporating new categories based on MRD, which should be measured either by NGF or NGS (or an equivalent validated technique) with a minimum sensitivity of 10^−5^ [109]. MRD status, whose independent prognostic value was confirmed in two different metanalyses [110,111], is now considered the most relevant predictor of clinical outcome, thus outweighing CR/sCR as the most valuable short-term therapeutic endpoint. Among patients in CR, indeed, improved PFS and OS have significantly been associated with undetectable MRD, regardless of cytogenetic risk, disease stage, or prior transplant [110]. Conversely, patients in CR, remaining MRD positive after therapy, have no better outcomes than patients in PR, as demonstrated by a pooled analysis of three PETHEMA/GEM clinical trials [112].

MFC is able to identify and quantify neoplastic PCs, whose atypical phenotype allows the distinction from normal PCs and from all other cell populations in different samples during the course of the disease [113]. Since the first demonstration of potential clinical relevance of MFC-MRD in MM [114,115], this method has progressively become one of the preferred tests for MRD assessment, because of its wide applicability (i.e., virtually in all MM patients) and availability, coupled with high specificity and sensitivity (i.e., ≤10^−4^). Most relevant markers used to identify and discriminate normal from aberrant PCs encompass CD38, CD138, CD45, CD56, CD19, CD27, CD28, CD81, CD117, and cytoplasmic k and λ light chains, combined within different gating strategies [113,116]. Normal PCs are most commonly CD38+ bright, CD138+ bright, CD81+ bright, CD45+, CD19+, CD27+, CD28−, CD20−, CD56−, CD117−, with a polyclonal cytoplasmic k/λ ratio [117]. Of note, small subsets (<30%) of normal PCs display less conventional antigen expression patterns, variably showing a diminished expression of CD45 and CD19, as well as aberrant expressions of CD20, CD28 and CD56 [118]. In contrast, neoplastic PCs exhibit several aberrancies, comprising multiple possible combinations of the followings: CD38 low (weaker than normal PCs), CD45−, CD19−, CD20+, CD27−, CD28+, CD81−, CD117+, CD56+, along with monoclonal cytoplasmic k or λ expression [116]. Notably, antigenic abnormalities are usually co-expressed within the same clonal PC population, whereas immunophenotypic variants observed in normal PCs are rather heterogeneous and/or distributed among distinct subsets [118]. In addition, the EuroFlow Consortium demonstrated that non-clonal PCs from either healthy donors or patients with non-PC-derived disorders display highly concordant immunophenotypes, not overlapping with those of >97% of MM-related PCs [2]. Moreover, the proneness of malignant PCs to major antigenic shift upon therapy is generally negligible [119], although small changes in phenotype (e.g., loss of CD56) might occur, because of clonal selection of chemo-resistant cells (antigenic restriction rather than shift) [120]. However, it should be noted that the treatment with anti-CD38 monoclonal antibodies (namely, daratumumab and isatuximab) undermines the reliability of this marker in the MRD assessment [121]. Therefore, new markers, such as CD200, CD33, CD54, CD229, CD307, CD319, CD150, and VS38, have been proposed to be included in MFC panels for identification of residual PCs (Table 1). Since the aberrant PC phenotype is usually specific in each individual patient and quite stable over time, antibody panels were historically built in a patient-specific fashion, requiring the presence of a diagnostic sample [115,122]. Afterwards, the development of fixed 4- to 7-color antibody panels covering most common MM-related abnormal phenotypes [108,119,123], alongside the release of the first consensus document by European Myeloma Network (EMN) [124], have allowed to step toward a more standardized MFC approach. Nonetheless, a systematically lower sensitivity (usually, by 1 log10) compared to molecular techniques and a lack of total uniformity in antibody panels, specific fluorochromes, and the number of cells to be analyzed constitute well-known limits of conventional MFC-MRD.

More recently, while NGS has replaced traditional molecular tests, novel MFC assays, endowed with a higher sensitivity (10^−5^–10^−6^), have superseded older flow methods. In particular, innovative EuroFlow-NGF constitutes a validated and standardized assay for highly sensitive (up to 10^−6^) and fast (<4 h) quantification of MRD in MM. This standardized approach, based on an optimized 2-tube 8-color (or, alternatively, single-tube 10-color) antibody panel, is applicable in 99% of MM cases and feasible in most laboratories [125]. EuroFlow-NGF comprises the use of an automatic population separator, allowing to eliminate the inter-operator variability, and a quality control of BM sample cellularity by simultaneous detection of B-cell precursors, erythroblasts, myeloid precursors, and/or mast cells [125]. This information is critical to ensure sample quality and identify hemodiluted marrow aspirates, which can lead to false negative results. Furthermore, the ability to perform a complete immune profile, including T, B, and NK cells, as well as macrophages and other myeloid cell populations, at the time of MRD assessment, could also be exploited to predict outcome in patients receiving immunotherapies, as “protective” immune profiles (consistent with an active immune surveillance) may account for prolonged survivals, observed in some cases, despite persistent MRD [126]. Finally, it has also been shown that the use of monoclonal anti-CD38 antibodies does not adversely affect MRD assessment in NGF. The high and independent prediction accuracy of EuroFlow-NGF, for both OS and PFS, was recently demonstrated in a Spanish prospective study including more than 400 newly diagnosed MM patients; in this large cohort, only 7% of patients without a detectable disease (reported sensitivity of 10^−6^) relapsed, predominantly with extramedullary disease [127]. Consistently, in the EMN02/HOVON 95 MM trial, patients with sustained MFC-MRD negativity (sensitivity of 10^−4^–10^−5^) had a 5-year PFS and OS of 81% and 94%, respectively, thus suggesting that MRD negativity can be used as a surrogate endpoint for survival outcomes [128].

Whilst MFC is generally considered less sensitive than sequencing (especially, NGS), to date, only a few studies have compared MRD results obtained with the two methodologies. The CASSIOPEIA trial reported a good concordance (83.5%, in paired samples) between NGS and NGF, both with a reported sensitivity of 10^−5^, in CR/sCR patients [129]. Moreover, in the FORTE study, second-generation MFC and NGS showed a good concordance (up to 86%) in CR/sCR patients, particularly when the same sensitivity was reached [130]. Similarly, a recent comparative analysis by Medina and colleagues reported an excellent level of agreement (86%) between NGF and NGS; in 10 out of 15 discordant cases, MRD positivity was missed by NGF [131]. In summary, the available evidence indicates that both NGF and NGS can virtually reach a sensitivity of 10^−6^, when specific technical conditions are met to reduce the false-negativity risk, related to the patchy nature of MM, occurring with both approaches. In other words, it could be argued that the ability to avoid false negatives due to lower detection limits, rather than the technique per se, is crucial to detect deeper levels of MRD. In this light, NGF is not intrinsically less sensitive than NGS, provided that an adequate number of cells (i.e., ≥2 × 10^7^), within a representative BM pool, are analyzed. In line with this notion, in one study of 274 paired PB/BM samples evaluated by NGF, MFC in PB was impressively less sensitive than in BM, as 40% of patients with BM residual disease had undetectable MRD in PB [132]. Therefore, according to an international consensus guidance recently released by an expert panel, PB-based MRD testing need to be further investigated and cross-validated with BM-based MRD assays [133].

Although several studies definitely demonstrated that MRD is a robust and reliable prognostic factor and, possibly, a surrogate endpoint enabling early interpretation of clinical trial results, the story of MRD in MM has just started. Several issues are still open, including: (i) patient selection (should MRD evaluation be restricted only to patients in CR/sCR or should be extended to patients in very good partial response as well?); (ii) optimal time points of MRD assessment during the treatment course (which is the best timing of evaluation? Is early evaluation useful?); (iii) optimal thresholds (10^−5^ or 10^−6^?); (iv) optimal technique(s) or combination of techniques to detect MRD both in BM and outside BM; (v) MRD-driven strategies (how can MRD guide the treatment in the individual patient?); (vi) frequency of MRD monitoring in MRD-negative patients; (vii) standard definitions of sustained MRD negativity and MRD recurrence following MRD negativity. As regards this latter point, IMWG has tentatively defined sustained MRD negativity as confirmation of NGS/NGF and positron emission tomography (PET) negativity after at least 1 year [109]. However, the concepts of stable MRD negativity and MRD relapse, as well as their respective clinical implications, need to be further refined, in order to enable MRD-driven therapeutic decisions.

In conclusion, by answering the aforementioned questions, it is expected to foster the employment of MRD in daily clinical practice, allowing a rational modulation of therapy on the basis of MRD.

## 8. Conclusions and Perspectives

The high prognostic significance of MRD detection has shown a remarkable impact on the therapeutic management of hematologic patients. MFC-MRD diagnostics offers the unique opportunity to phenotypically recognize the resistant/recurrent neoplastic cells, since their appearance at very low levels in the BM and, possibly, also in the PB. Today, standardized MFC for MRD monitoring constitutes a fundamental strategic tool, complementary to molecular MRD assessment, for risk-adapted therapy decision-making, in several hematologic settings (Table 1). To date, MRD-driven therapy intensification has become a mainstay in the management of acute leukemias, while the application of this kind of “pre-emptive” strategy to chronic hematologic neoplasms still needs further investigations. In particular, it remains to be better assessed to what extent the detection of positive MRD can induce opportune therapeutic modifications, or whether stable low-level disease could be considered a sufficient therapeutic achievement for some subsets of patients (e.g., frail patients) with CLL or MM [134,135].

Of note, MFC-MRD approach is essential to investigate expression levels and clonal patterns of specific surface antigens that can be targeted by novel immunotherapies (e.g., monoclonal antibodies and CAR-T cells). Compared to molecular MRD methods, MFC-based MRD detection currently remains slightly less sensitive, but more rapid and applicable in the vast majority of patients, with a valuable profile in terms of cost-effectiveness in everyday clinical practice. However, high throughput “next-generation” approaches are expected to overcome actual biological and technical issues of both MFC and molecular MRD methods, providing very high sensitivity (<10^−6^) and applicability (>99%), as well as in-depth phenotypical and genetic characterization of the neoplastic clone (Figure 1). Notwithstanding, the routine use of MFC-MRD is typically hindered by technical challenges and interpretative complexities, making still advisable to centralize such analyses in experienced laboratories. Indeed, flow cytometry still relies heavily on the expertise of operators, who must take into account pre-analytical bias and variability in instruments, fluorochromes, analysis software, and individual antigens. Progressive introduction of automated cytometric evaluations by using highly specialized software should significantly help to improve the standardization of such complex multiparametric analysis. 

Progressive technological advances in MFC-MRD measurement are raising new challenges regarding the correct clinical use of information on extremely low levels of MRD: with differences among various hematologic settings, the biological significance of such MRD levels and the related kinetics of relapse, as well as its optimal clinical management, are still largely unknown. It is conceivable that some relevant immunological factors may directly be implied in such “pre-clinical” tumor progression, in line with the general notion that most if not all neoplasms are under immune control and have to escape host immunity to outgrow. In this view, contextually to MRD detection, the MFC-based approach could also allow to investigate the frequencies and functionality of tumor-specific T cells, which are emerging as a fundamental protective factor against neoplastic outgrowth, both in hematologic and solid cancers [56,57,58,136,137,138,139,140,141].

**Figure 2:**
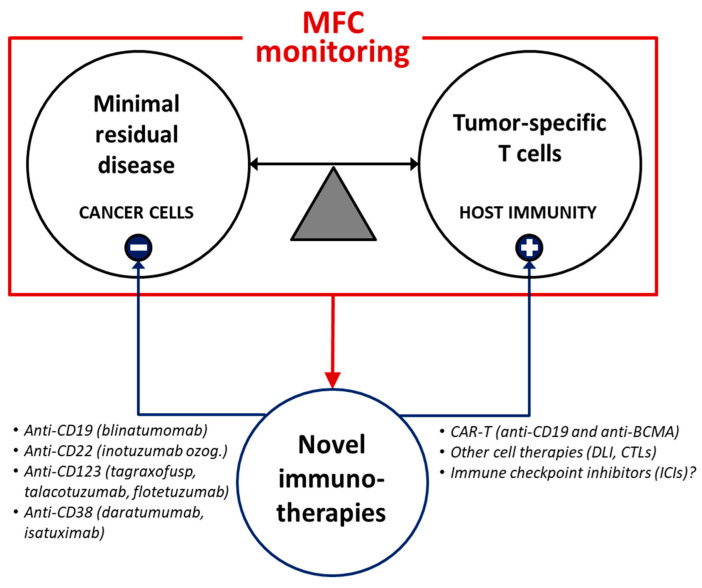
MFC applications may guide early immunotherapeutic strategies during the follow-up of hematologic malignancies. Advanced MFC-based monitoring of both MRD and protective immunity may contribute to the rational use of innovative target therapies (such as monoclonal antibodies, cell therapies and other immunomodulatory treatments). DLI = donor lymphocyte infusion. CTLs = cytotoxic T lymphocytes. BCMA = B cell maturation antigen.

Further collaborative works within international networks are warranted to (i) improve the standardization of MFC-based clinical-laboratory protocols, (ii) better combine cytometric and molecular MRD monitoring in the clinical management of hematologic neoplasms, and (iii) adequately exploit the potentials of MFC diagnostics to develop and guide novel therapeutic strategies. In perspective (Figure 2), the promising integration between advanced MRD diagnostics, antitumor T cell monitoring and innovative target treatments may allow to seek and eliminate both quiescent cancer stem cells and other neoplastic subclones escaping host immunosurveillance, hence paving the way toward full personalized medicine for hematologic patients.

## Figures and Tables

**Table 1:** Main features of MFC-based MRD monitoring in acute leukemias, CLL and MM.

	AML	B-ALL	T-ALL	CLL	MM
**Sensitivity**	10^−3^–10^−5^	10^−4^–10^−5^	10^−4^–10^−5^	10^−4^–10^−5^	10^−5^–10^−6^
**Sample origin**	BM	BM	BM, PB	PB, BM	BM
**N° of cells required**	3 × 10^6^	4 × 10^6^	4 × 10^6^	3 × 10^6^	5–20 × 10^6^
**Applicability (% of cases)**	>97%	>99%	>99%	>95%	>99%
**MRD “positivity”** **threshold**	≥10^−3^	≥10^−4^	≥10^−4^	≥10^−4^	≥10^−5^
**Follow-up timepoints**	Poorly standardized: usually performed early, after initial therapy (i.e., post-induction/consolidation treatments), then usually guided by clinical protocols (usually, every 3–6 months)				
**Backbone panel**	CD34, CD117, CD45, CD13, CD33, CD15, CD7	CD34, CD19, CD10, CD20, CD38, CD45	CD2, CD3, CD5, CD7, CD4, CD8, CD34, CD45, CD99, CD1a	CD19, CD20, CD5, CD79b, CD43, CD81	CD138, CD38, CD45, CD56, CD19, CD27, CD28, CD117, cy k/λ, CD81
**Additional markers**	CD14, CD64, HLA-DR, CD4, CD11b, CD123, CD133, CD38, CD90	CD22, CD81, CD66c, CD123, CD73, CD304	CD10, CD38, CD56, TdT	CD200, CD23, CD160, ROR1	CD33, CD54, CD200, CD229, CD307, CD319, CD150, VS38
